# Trajectories of distress and recovery, secondary stressors and social cure processes in people who used the resilience hub after the Manchester Arena bombing

**DOI:** 10.1192/bjo.2023.527

**Published:** 2023-08-08

**Authors:** John Stancombe, Richard Williams, John Drury, Louise Hussey, Matthew Gittins, Alan Barrett, Paul French, Prathiba Chitsabesan

**Affiliations:** Young People's Mental Health Research Unit, Pennine Care NHS Foundation Trust, UK; Welsh Institute for Health and Social Care, University of South Wales, UK; School of Psychology, University of Sussex, UK; National Institute for Health Research Applied Research Collaboration Greater Manchester, Health Innovation Manchester, UK; and School of Health Sciences, University of Manchester, UK; Centre for Biostatistics, University of Manchester, UK; Manchester Resilience Hub, Pennine Care NHS Foundation Trust, UK; and School of Health Sciences, University of Salford, UK; Research and Innovation Department, Pennine Care NHS Foundation Trust, UK; and Faculty of Health, Psychology and Social Care, Manchester Metropolitan University, UK

**Keywords:** Trajectories of distress, Manchester Arena bombing, secondary stressors, trajectories of recovery, social support

## Abstract

**Background:**

Terrorist incidents lead to a range of mental health outcomes for people affected, sometimes extending years after the event. Secondary stressors can exacerbate them, and social support can provide mitigation and aid recovery. There is a need to better understand distress and mitigating factors among survivors of the Manchester Arena attack in 2017.

**Aims:**

We explored three questions. First, what experiences of distress did participants report? Second, how might secondary stressors have influenced participants’ psychosocial recoveries? Third, what part has social support played in the relationships between distress and participants’ recovery trajectories?

**Method:**

We conducted a cross-sectional online survey of a convenience sample of survivors of the Manchester Arena bombing (*N* = 84) in January 2021 (3 years 8 months post-incident), and a longitudinal study of the same participants’ scores on mental health measures over 3 years from September 2017.

**Results:**

Survivors’ mental well-being scores in early 2021 were significantly lower than general population norms. Longitudinal follow-up provided evidence of enduring distress. Secondary stressors, specifically disruptions to close relationships, were associated with greater post-event distress and slower recovery. We found an indirect relationship between identifying with, and receiving support from, others present at the event and mental well-being >3 years later.

**Conclusions:**

The Arena attack has had an enduring impact on mental health, even in survivors who had a mild response to the event. The quality of close relationships is pivotal to long-term outcome. Constructive support from family and friends, and people with shared experiences, are key to social cure processes that facilitate coping and recovery.

In the UK, major incidents are defined as ‘an event or situation with a range of serious consequences that require special arrangements to be implemented by one or more emergency responder agency’.^1^ They lead to a range of mental health outcomes that extend months and, sometimes, many years.^2^ Distress during and after incidents is ubiquitous, but not necessarily a function of psychopathology, including mental illness.^3–5^

## The nature of distress

A term that requires greater clarity is ‘distress’. There are three common approaches. An epidemiological approach is based on literature that refers to distress being composed of subthreshold symptoms of anxiety, depression or post-traumatic stress disorder.^6^ A typological approach uses the term in relation to emergencies to depict people who have a range of experiences that are anticipated, and usually broader than symptoms of common mental disorders. Some accounts organise these experiences into emotional, cognitive, social and physical domains.^7^ A third approach is based on the experiences reported by people who say that they have been or are subjectively distressed.^8^ Our definition recognises that people are likely to feel stressed in emergencies. Their experiences are described as distress when their stress is accompanied by emotions, behaviours, thoughts and physical sensations that are upsetting or affect their relationships and functioning. Distress is not a diagnosis, but may accompany a disorder.

## Trajectories of distress and recovery after major incidents

There are many models of the risk of mental health disorder in the aftermath of major incidents.^9–11^ However, relatively little is known about the spectrum and course of distress apart from the broad categories or trajectories of response.^12^ Research has been dominated by biomedical models addressing epidemiological issues and identification and treatment of psychiatric disorders; there has been less focus on the majority of people who experience distress that does not meet the threshold for specialist services.^13^ There is a need to better understand their experiences, using measures of distress that are less focused on symptoms of disorders and more sensitive to the broader range of experiences.

## Primary and secondary stressors

The psychosocial effects of extreme events can be influenced by a complex combination of primary and secondary stressors. Primary stressors are factors inherent in particular major incidents, disasters and emergencies and arise directly from them. Secondary stressors are defined as social factors and people's life circumstances that exist before, and affect people during, the major incident; and/or societal and organisational responses to an incident or emergency.^14^ Examples include breakdown of family relationships, lack of support in people's workplaces, concerns about access to appropriate healthcare and overwhelming workloads.

Secondary stressors affect people's well-being and mental health in ways that exacerbate the direct effects of major incidents.^14,15^ They are potentially tractable and should be included in psychosocial care programmes to mitigate distress and mental health disorders. However, our current understanding of the role of secondary stressors in the context of terrorist attacks has been limited.

## Social cure processes

Disasters happen to people collectively. One factor that can mitigate both primary and secondary stressors are new group relationships and any associated social support. Social support can contribute to survivors’ well-being,^16,17^ and reduce psychiatric symptoms.^18–20^ The support that people receive from their families, friends, colleagues, organisations and communities has a profound effect on the meaning people derive from events, their feelings of control, their agency and their capacity to deal with adversity.^16,17^

Offers of support may be experienced differently depending on who they come from,^21^ with certain forms of social support experienced as unhelpful by survivors.^22,23^ Recent studies of a variety of emergencies have found that survivors reported particular benefit from support from people who shared the experience of the emergency with them.^24–26^ They see these people as understanding their distress and being willing to listen. In our qualitative study, we found that many survivors felt constrained from sharing their feelings with friends and families if survivors perceived them as unable to understand their experiences.^13^ They also described various forms of helpful social support, including social validation, which was a feature of support provided by others.

Access to groups based on shared experience is an important factor for many people in their coping and recovery, and a possible springboard to personal growth.^27^ These common experiences can create a new shared social identity based on the category of people involved in the incident.^28^ Sharing social identity motivates people to give support to others and expect support from them. Shared social identity and perceived support also increase the sense of group efficacy, defined as the perceived ability to coordinate, and respond collectively to the disaster.^28,29^ This approach, examining social identity as a mechanism for well-being, is known as the social cure.^29^

## Our study

The study reported here is part of the two-phase, mixed-methods, exploratory and co-productive research programme Social Influences on Recovery Enquiry (SIRE) (www.penninecare.nhs.uk/services/types/manchester-resilience-hub/public-information). Phase 1 involved qualitative analysis of semi-structured interviews with 18 survivors of the Manchester Arena attack, to characterise their experiences and opinions on the psychosocial care that affected their recovery.^13,27^ Informed by findings from phase 1, phase 2 is an exploratory quantitative analysis that complements our earlier qualitative study and tests whether our interviewees’ experiences were replicated in a larger sample. Our semi-structured interviews and the questions in the online survey were developed with survivors. We used learning points from analysis of the qualitative interviews to generate questions for our online survey. Thus, we constructed bespoke measures, grounded in the experiences of our interviewees.

We sought to answer three questions. First, what experiences of distress did each participant report, and how did this change over time? Based on our qualitative findings,^13^ we expected distress to be universal and enduring for the majority, with people who had milder reactions being more likely to show improvement over time. Second, how might secondary stressors have influenced participants’ psychosocial recoveries? Based on our previous study,^13^ we anticipated participants’ experiences of secondary stressors would correlate with heightened distress and low well-being. We also compared the impact of different types of secondary stressor on participants’ distress trajectories. Finally, what part have social cure processes, in particular social identity and use of social support, played in the relationships between distress and participants’ recovery trajectories? Based on our qualitative study and previous research,^27,28^ we expected support from families, friends and people who were at Manchester Arena to be perceived as more helpful than support from other sources. We anticipated that support measures would be associated negatively with distress and positively with current mental well-being.

This paper presents the findings from our bespoke survey and from a longitudinal analysis of validated questionnaires completed for the Manchester Resilience Hub. The Hub was established following the Arena bombing in 2017 as a National Health Service (NHS) remote consultation service to help people from across the UK who were experiencing problems resulting from the incident. It provided a central point for psychosocial support, mental health advice and an online monitoring programme to identify people who might require specialist mental health or other services.^30^ This paper develops our previous findings by examining further the interrelationships between social support, secondary stressors, post-event distress and mental health outcomes among physically uninjured survivors of the Arena attack.

We compared two different pathways by which social identification could operate to reduce distress and facilitate recovery. In model 1, social identity is the basis of perceived support and hence efficacy.^19,31^ In model 2, perceived support is the basis for seeing others as part of their ingroup and identifying with them.^27^ In both models, efficacy is expected to be strongly associated with well-being. We report testing of how these models apply to our data from a sample of survivors, assessed through the significance of indirect pathways.

## Method

### Design

The present study is a quantitative analysis of two data-sets provided by the same participants. The first data-set is a cross-sectional online survey of a convenience sample of survivors of the Manchester Arena attack (*N* = 84), completed in January 2021 (3 years 8 months post-incident). This includes a measure of participants’ retrospective experiences of distress in the 3 months after the event, using an unvalidated scale that is based on analysis of the subjective accounts of survivors who took part in our earlier qualitative study.^13^ It also includes a validated mental well-being scale (Warwick–Edinburgh Mental Wellbeing Scale; WEMWBS). This measures participants’ mental health in terms of the broad categories of psychological functioning, life satisfaction and ability to develop and maintain mutually benefiting relationships, administered at a time point nearly 4 years after the event.

The second data-set, the longitudinal element of our study, is the same participants’ contemporaneous scores on self-report mental health questionnaires collected prospectively online, from 3 months (September 2017) to 3 years post-incident (last data point was September 2020). We use the results of validated mental health measures to define the levels of distress experienced by participants over the course of 3 years following the attack.

The differing yet complementary perspectives and the cross-sectional and longitudinal analyses enabled exploration of participants’ distress trajectories, and how secondary stressors and social cure processes might have affected participants’ coping and recovery.

### The sample

The study was conducted with registrants of the Manchester Resilience Hub. They were survivors of the Arena attack who contacted the service and completed the mental health questionnaires used at the Hub. Within a year of the attack, the Hub had registered over 3000 people, representing approximately 16% of people who were physically present at the Arena during the attack.^32^ The participants who completed the SIRE online survey (*N* = 84) were recruited from a convenience sample of research-willing registrants (*N* = 262). Eleven (13%) of the survey sample had also participated in the phase 1 qualitative interview study.

### Eligibility criteria

Eligible participants were identified through the Hub database and were people who (a) attended and were directly affected by the Arena event, but were not physically injured; (b) completed the Hub's measures of mental health at the 3 and/or 6 month post-event time points; (c) were aged ≥18 years on registration with the Hub and (d) had given consent to be contacted about participation in research.

### Measures

#### Cross-sectional survey measures

The majority of our survey questions are unvalidated items based on the subjective experiences of the participants in our previous interview study. Our rationale was to substantiate and amplify our earlier findings. We did not intend to create and standardise a new scale for further research or clinical use. A full copy of the questionnaire, comprising 103 items, and items not included in the analyses presented here, are given in the Supplementary Material available at https://doi.org/10.1192/bjo.2023.527.

##### Measures of well-being and distress

We included a single item measuring stress and distress in the month before the incident, and asked participants to choose which of the options best described their experiences.

We measured well-being with the WEMWBS. The WEMWBS is a 14-item, psychometrically robust measure of current mental well-being in the general population, validated in a number of populations, including clinical and ethnic minority samples.^33,34^ It covers feeling and functioning aspects of mental well-being, which it represents as the positive end of a continuum. The scale is 1 (none of the time) to 5 (all of the time) (*α* = 0.94).

To measure post-event distress, we constructed 29 items aimed to give a measure of participants’ experiences in the first 3 months after the event. The items were based on the experiences of distress reported by the participants in our earlier interview study.^13^ All of the questions were scored from 1 (not at all) to 5 (all of the time) (*α* = 0.97).

To measure secondary stressors, we constructed 18 items, drawn from the findings from the qualitative phase of our study, to measure difficult or stressful things that sometimes happen to people before, during and after major events.^13^ Participants were asked to rate how frequently since the event they had experienced stress with each item. Questions were scored from 1 (never) to 5 (a great deal) (*α* = 0.92).

##### Social cure variables

To assess identification with others who shared the same experience at the Arena attack, we used three items, adapted from the items in Leach et al,^35^ to assess participants’ identification with other people with direct experience of the Arena attack (*α* = 0.89). The validity and reliability of these items have been established in a broad range of social groups.^36^

Based on established measures used in previous research studies,^20^ we developed three items to measure personal and group efficacy in the domain of coping after the attack (*α* = 0.70). We also included one item measuring efficacy in accessing services.

To assess perceived social support, we constructed 21 items that covered sources and types of support that were based on the experiences of social support reported by the participants in our earlier interview study.^27^ They included nine items referring to perceived support from others who were at the Arena attack (*α* = 0.82), three items referring to perceived support from family and friends who were not at the Arena attack (*α* = 0.80), three items referring to perceived support from people at one's workplace (*α* = 0.89) and one item referring to perceived support from professionals.

#### Longitudinal Hub measures

Survivors completed four questionnaires online when registering with the Hub. Three measure symptoms of mental health disorders (the Trauma Screening Questionnaire (TSQ),^37^ Patient Health Questionnaire-9 (PHQ-9)^38^ and Generalised Anxiety Disorder-7 (GAD-7)^39^). The fourth, the Work and Social Adjustment Scale (WSAS), measures functional impairment.^40^ These measures are validated and have established clinical cut-off points. The Hub identified clinical priority with this online tool, supplemented by telephone contact from a clinician, if indicated. The initial assessment took place 3 months after the attack (September 2017), and survivors were invited to repeat the online measures at regular intervals thereafter (6, 9, 12, 18, 24, 30 and 36 months post-event).

### Procedure

Eligible participants for the online survey were invited to participate by email (using the NHS mail encryption service to ensure security of data) and supplied with the patient information sheet. The survey data were collected online with SNAP version 11 for Windows (SNAP Surveys, Bristol, UK; see https://www.snapsurveys.com/survey-software/), a GDPR-compliant, cloud-based platform. The survey items were scored on Likert scales to measure how initial experience of distress and social and contextual factors have affected mental health, coping and recovery. Participants’ longitudinal scores on the triage measures were obtained from the Hub database, with their consent.

### Consent and ethical approval

Participants were given the legally required data protection and participant information in a retainable form. The online questionnaire included a final page containing a ‘submit’ button, prefaced by a statement reminding each participant that clicking the final button would constitute that participant giving informed consent in full knowledge of the data protection and participant information provided. Participants were able to withdraw from the survey without explanation at any point.

Ethical approval was provided by the UK's Integrated Research Application System process (application 255819).

### Data analysis

The results of the Hub's four measures were used to provide an overall categorical rating of mild, moderate and severe responses for each participant, at first completion and over the course of the 3 years following the attack. This categorisation was based on the scoring algorithm developed by the Hub, based on validated clinical thresholds, to group people by severity into clinical priorities.

- Mild response: People in this subgroup (*n* = 42) had initial scores of TSQ < 6, PHQ-9 = 0–9, GAD-7 = 5–9 and WSAS = 0–10.
- Moderate response: People in this subgroup (*n* = 19) had initial scores of TSQ = 6, and/or PHQ-9 = 10–19 or 1 on the self-harm item, and/or GAD-7 = 10–14 and/or WSAS = 11–20.
- Severe response: People in this subgroup (*n* = 23) had initial scores of TSQ ≥ 6 and/or PHQ-9 = 20–27 or ≥2 on the self-harm item, and/or GAD-7 ≥ 15 and/or WSAS ≥ 21.

We examined the descriptive statistics for the questions in the cross-sectional survey for differences between the three response groups, using parametric and non-parametric tests as appropriate. With participants’ consent, these data were also linked with clinical information held in health records at the Hub. Thus, we examined the extent to which factors measured in our survey predicted participants’ coping and recovering, including the extent to which the effects of social support are mediated by shared experience and social identity.

A post-event distress score was derived for each participant by calculating the mean of all of their scores on the 29 questions intended to measure post-event distress. Other variables were constructed by calculating their mean scores on the secondary stressor and social cure items within the survey. Each participant's WEMWBS score was derived by summing all of the items within the scale giving a potential range of 14–70. It provides a measure of participants’ mental health at the time when they completed the survey in late 2020 and early 2021.

We carried out regression analysis, analysis of variance (ANOVA), mediation analysis and path analysis to generate the inferential statistics used to address the study questions.

## Results

### Demographics

Of the 262 people who met our eligibility criteria, 84 (32%) responded to the survey. We were unable to use the longitudinal data from all 262 people because we did not have permission to use clinical data from all but the 84 people who did consent.

The Supplementary Material includes a demographic breakdown of our survey respondents (Supplementary Table 1). There were no significant differences in gender (*χ*^2^ = 1.34, *P* = 0.247) between the survey respondents (*N* = 84) and all Hub registrants. The age profile of our survey respondents was skewed toward older age compared with all Hub registrants (*χ*^2^ = 9.17, *P* = 0.027). The majority of respondents described themselves as ‘White British’ (*n* = 78, 93%), which closely corresponds with ethnicity data recorded on the Hub database (94% ‘White British’). A total of 47% were from North-West England (including 21% from Greater Manchester), which is representative of all Hub registrants.

### What were participants’ experiences and trajectories of distress and recovery over time?

#### Experiences of distress

We examined all of the post-event distress questions and differences between the mean for each item and the overall mean for all of the items combined (3.40) (Supplementary Table 2). Feeling ‘unusually alert or on my guard’ (4.35) and ‘shocked’ (4.17) were the highest scoring items, followed by ‘worries about the risk of another terrorist attack’ (4.12) and ‘upsetting thoughts or images about the event’ (4.12). The lowest scoring items were being ‘unsure about where I was or about what was the date’ (2.36) and ‘losing skills that I had before the event’ (2.40).

#### Current mental well-being

The WEMWBS mean score for our sample is 42.4 (s.d. 9.9) with a median of 43 (interquartile range 35–51). Table 1 shows the descriptive statistics of the survey respondents on WEMWBS compared with the Health Survey for England 2011 data.^33^

**Table 1:** Descriptive statistics for the Warwick–Edinburgh Mental Wellbeing Scale scores

	England	Social Influences on Recovery Enquiry survey participants^a^		
		Both quantitative survey and qualitative interviews (*n* = 11)	Quantitative survey only (*n* = 73)	All survey participants (*N* = 84)
*N*	7020	11	73	84
Mean	51.67	47.09	41.74	42.44
Mean difference		4.58	9.96	9.26
95% CI		−0.58 to 9.74	7.95–11.97	7.39–11.13
Median	53	47	42	43
s.d.	8.71	6.96	10.21	9.98
Minimum	14	33	20	20
Maximum	70	57	65	63
Interquartile range		10	16	16
Skewness	−0.66	−0.54	−0.07	−0.18
Kurtosis	1.22	0.13	−0.64	−0.6

a. Eleven (13%) of the survey sample also participated in the phase 1 interview study. The difference between the mean scores of this subgroup and the remainder of the sample (*n* = 73) fell short of significance (*t* = 1.744, d.f. = 7029, *P* = 0.0812).

The mental well-being of our participants was lower than the general population (*P* < 0.0001), with a mean difference of 9.3 (95% CI 7.4–11.1). A total of 46% of our sample scored below the 15th centile.

#### Post-event distress and current mental well-being

The relationship between current mental well-being (WEMWBS score) and our participants’ reports of post-event distress was examined with the Pearson correlation coefficient. The overall association between WEMWBS scores and the combined score for all of the post event distress items was low (*r* = −0.170, *P* = 0.123). Only four items had both significant association and mild-to-moderate effect sizes: question 33, ‘I wanted to be by myself most of the time’ (*r* = −0.300, *P* = 0.006); question 34, ‘I was irritable without good reason and took it out on other people or things’ (*r* = −0.252, *P* = 0.021); question 35, ‘I had serious disagreements or arguments with other people that were unusual for me’ (*r* = −0.246, *P* = 0.024) and question 22, ‘I had problems remembering things’ (*r* = −0.226, *P* = 0.038).

##### Exploratory factor analysis of post-event distress items

We conducted a principal axis factor analysis on the 29 post-event distress items, to explore the extent to which they made up different factors. Five factors had eigenvalues over Kaiser's criterion of 1, and together explained 64% of the variance in post-event distress scores. However, the first factor alone explained 51% of the variance and had by far the largest eigenvalue. The scree plot also indicated only one factor. The factor loadings of the post-event distress items after rotation are shown in the pattern matrix (Supplementary Table 3). Factor 1 contains five items, which appear to measure social withdrawal/physical symptoms (e.g. ‘I had persistent physical symptoms that I did not have before the event’, ‘I wanted to be by myself most of the time’). Factor 2 contains five items that appear to measure fear of recurrence (e.g. ‘I was very worried about the risk of another terrorist attack’). Factor 3 contains seven items that index impaired everyday functioning (e.g. ‘I had problems remembering things’). Factors 4 and 5 each contain five items, which appear to measure changes in affect (e.g. ‘I felt shocked’) and intense feelings (e.g. ‘I had angry outbursts’), respectively.

##### Regression analysis (WEMWBS and post-event distress factors)

We examined the extent to which the five post-event distress factors predicted WEMWBS scores (Table 2). They explained 16% of the variance in WEMWBS scores. The social withdrawal/physical symptoms factor was not predictive of mental well-being at 3 years 8 months post-incident. Impaired everyday functioning and change in affect were predictive factors. The beta-coefficients indicate that the magnitude of effect of each factor varied from small to moderate.

**Table 2:** Regression analysis of Warwick–Edinburgh Mental Wellbeing Scale scores against five post-event distress factors

		Unstandardised coefficients		Standardised coefficients			Collinearity statistics	
Post-event distress factor		B	s.e.	*β*	*t*	Significance, *P*-value	Tolerance	Variance inflation factor
1	Social withdrawal/physical symptoms	1.158	1.521	0.110	0.761	0.449	0.556	1.799
2	Fear of recurrence	−0.204	1.570	−0.020	−0.130	0.897	0.500	2.000
3	Impaired everyday functioning	3.777	1.573	0.369	2.401	0.019*	0.489	2.045
4	Changes in affect	3.563	1.589	0.334	2.242	0.028*	0.520	1.925
5	Intense feelings	−2.613	1.438	−0.252	1.818	0.073	0.600	1.668

*P<0.05

#### Longitudinal Hub measures

##### Response category at initial assessment

Based on the Hub algorithm, participants' scores on the Hub measures at 3 months (83 participants) or 6 months (one participant) were categorised as mild (*n* = 42, 50%), moderate (*n* = 19, 23%) or severe (*n* = 23, 27%) responses. Comparison of initial assessment data for all Hub registrants with the data for survey responders showed that there were differences between the two groups on two of the Hub's measures (the PHQ-9 and WSAS) (Supplementary Table 4). More people were categorised with severe reactions in the survey group.

##### Changes in Hub scores over time

Longitudinal analysis of the Hub scores of our survey respondents showed that there were decreases in their GAD-7 and TSQ scores over 3 years (Table 3). However, the beta-values and confidence intervals associated with these changes indicate small effect sizes. The PHQ-9 and WSAS scores did not decrease over 3 years.

**Table 3:** Changes in Hub scores over 3 years for survey responders

Screening score	Pearson *r*	*R*^2^	*β*	Lower 95% CI	Upper 95% CI	Significance, *P*-value
Patient Health Questionnaire-9	−0.061	0.004	−0.184	−0.455	0.087	0.183
Generalised Anxiety Disorder-7	−0.093	0.009	−0.25	−0.493	−0.007	0.043
Trauma Screening Questionnaire	−0.162	0.026	−0.211	−0.328	−0.094	<0.001
Work and Social Adjustment Scale	0.009	0	0.038	−0.062	0.138	0.454

**Fig. 1:**
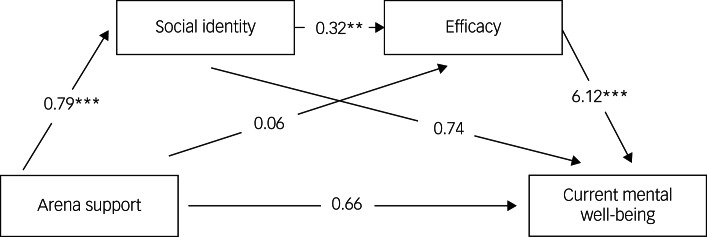
Serial mediation model with social identity and efficacy as mediators of the relationship between Arena support and current mental well-being (model 2) (**P < 0.01, ***P < 0.001).

Linear regression analysis showed (Supplementary Table 5 and Fig. 1) that there were differences, albeit small effect sizes, between the mild, moderate and severe distress response groups in the trajectory of scores on the Hub measures over 3 years. The mild response group showed decreases in GAD-7 (*R*^2^ = 0.036, *P* = 0.002) and TSQ (*R*^2^ = 0.083, *P* < 0.001) scores from the 12-month time point. The moderate response group showed reductions in PHQ-9 (*R*^2^ = 0.073, *P* = 0.014) and TSQ (*R*^2^ = 0.071, *P* = 0.018) scores from the 18-month time point. In contrast, the severe response group did not show any reduction in scores on any of the four Hub measures. None of the three response groups showed any improvement in WSAS scores over time. Of note, the TSQ was the only Hub measure at initial assessment (3 or 6 months) that did not correlate with final distress response categorisation (30 or 36 months).

##### Changes in each participant's response category over time

We compared the Hub initial (3 or 6 months) and final response categories (30 or 36 months) to give an indication of respondents’ improvement or deterioration over time (Table 4). Twenty-six of the 84 (31%) survey respondents did not have Hub scores at either 30 or 36 months; therefore, this analysis is based on 58 (69%) participants. Table 4 shows the number of participants who changed category between the initial and the final assessment. Overall, 13 (22%) respondents improved, 41 (71%) stayed the same and four (7%) deteriorated; 22 of the 58 (38%) respondents were still experiencing moderate or severe distress at 30–36 months.

**Table 4:** Changes in response categorisation between initial and final measurements based on the hub criteria

Overall response category (based on Hub scores)		Change	Total
Initial (3 or 6 months)	Final (30 or 36 months)		
Mild	Mild	No change	27
Mild	Moderate	Deteriorate	2
Mild	Severe	Deteriorate	0
Moderate	Mild	Improve	4
Moderate	Moderate	No change	6
Moderate	Severe	Deteriorate	2
Severe	Mild	Improve	5
Severe	Moderate	Improve	4
Severe	Severe	No change	8
Total			58

##### WEMWBS and post-event distress scores and their correlation with Hub scores

Supplementary Table 6 shows the correlations between the WEMWBS and post-event distress scores with the Hub scores at 3, 6 and 36 months after the incident. There were high correlations between some of the initial (3 or 6 month) Hub assessment measures and WEMWBS (completed >3 years later). This was most significant for the WSAS, followed by the PHQ-9. The GAD-7 was significant at 3 months, but not at 6 months. The TSQ scores at 3 and 6 months were not associated with participants’ mental well-being after 3 years 8 months. Of note, the contemporaneous Hub scores at initial assessment (3 or 6 months) have high correlations with our retrospective measure of post-event distress.

### How might secondary stressors have influenced participants’ psychosocial recoveries?

#### Experiences of secondary stressors

We examined all of the secondary stressor items for differences between the mean for each one and the overall mean for all of the items combined (Supplementary Table 7). The most commonly reported stressors were ‘exposure to negative reports in the news media’, ‘new or continuing mental health problems’ and ‘social media’, causing a ‘moderate amount’ or a ‘great deal’ of stress to 62%, 60% and 54% of our respondents, respectively. The lowest scoring and less frequently reported stressors were ‘lack of access to physical healthcare’ (8%), ‘lack of education opportunities or facilities’ (11%) and ‘loss or lack of employment’ (15%).

#### Secondary stressors and current mental well-being

The relationship between current mental well-being (WEMWBS score) and the secondary stressor items was examined with the Pearson correlation coefficient. The overall correlation between WEMWBS score and the combined score for all of the secondary stressor items was moderate (*r* = −0.263, *P* = 0.016). The only secondary stressors with significant associations and moderate effect sizes were ‘disruption to relationships with friends’ (*r* = −0.353, *P* = 0.001), ‘disruption to relationships with my family’ (*r* = −0.318, *P* = 0.003) and ‘new or continuing mental health problems’ (*r* = −0.308, *P* = 0.004).

##### Exploratory factor analysis of secondary stressor items

We conducted a principal axis factor analysis on the 18 stressors to explore the extent to which the secondary stressor items clustered. Five factors had eigenvalues over Kaiser's criterion of 1 and, in combination, explained 65% of the variance. The scree plot did not clearly suggest five and indicated two (Supplementary Table 8 shows the factor loadings after rotation). The items that cluster on the same factor suggest that factor 1 represents family and friends stressors (e.g. ‘disruption to relationships with my friends/within my family’), factor 2 represents work stressors (e.g. ‘loss or lack of employment’), factor 3 represents compensation stressors (e.g. ‘difficulties with making an application to the compensation scheme’), factor 4 represents service stressors (e.g. ‘difficulties with finding information and support’, ‘lack of access to the mental healthcare I need’) and factor 5 represents physical health stressors (e.g. ‘new or continuing physical health problems’). Family and friends was the only secondary stressor factor that had a significant correlation with WEMWBS score. The magnitude of effect was moderate.

##### Multiple regression analysis

The five secondary stressor factors were put into a regression equation with WEMWBS score as the dependent variable (Table 5). The secondary stressors together explain approximately 17.5% of the variance of WEMWBS score, but only one emerges as significant. The family and friends factor accounts for the majority of the association, with a moderate effect size (*β* = −0.369); the other factors were not predictive of WEMWBS score.

**Table 5:** Regression analysis of Warwick–Edinburgh Mental Wellbeing Scale scores and five secondary stressor factors

	Unstandardised coefficients		Standardised coefficients	*t*	Significance, *P*-value
	B	s.e.	*β*		
Family and friends	−3.942	1.150	−0.369	−3.429	0.001
Work	0.264	1.130	0.025	0.234	0.816
Compensation	−0.668	1.125	−0.064	−0.594	0.554
Services	1.373	1.181	0.125	1.163	0.249
Physical health	−1.543	1.102	−0.150	−1.400	0.166

#### Longitudinal Hub scores and secondary stressors

One-way ANOVA was carried out to compare the overall mean of participants’ secondary stressor scores across the three different response categories (mild, moderate and severe), based on the Hub measures (Supplementary Table 9). Participants’ mean scores on the secondary stressor items were significantly correlated with response category at both initial (*F* = 13.35, *P* < 0.001) and final (*F* = 7.35, *P* < 0.001) assessment. *Post hoc* comparison, using Tukey's honestly significant difference to correct for type 1 errors, showed that there were higher scores for secondary stressors for people in the moderate and severe response groups compared with the mild response group at both the initial (mild versus moderate: mean difference 0.527, 95% CI 1.007–0.0474; mild versus severe: mean difference 1.006, 95% CI 1.478–0.533) and final (mild versus moderate: mean difference 0.562, 95% CI 1.110–0.0139; mild versus severe: mean difference 0.836, 95% CI 1.424–0.248) assessment. Hence, secondary stressors were not only associated with the relative severity of people's initial reactions, but also with the enduring nature of those reactions.

### What part have social cure processes played in participants’ recoveries?

#### Experience of social support

We examined all of the questions constituting the social cure variables (identification with others who shared the same experience at the Arena, efficacy and perceived social support) and their association with post-event distress, secondary stressors, distress trajectory and current mental well-being (WEMWBS score).

The perceived social support variable comprises items from three different sources: others who were at the Arena attack, family and friends who were not at the Arena attack and people's workplaces. Thus, in total, five social cure items were subject to analysis: identification with others at the Arena (named ‘social identity’), efficacy, support from people at the Arena (named ‘Arena support’), support from family and friends not at the Arena and support from people at one's workplace.

#### Comparison of sources of social support

Repeated measures ANOVA was carried out to compare participants’ responses across the three different sources of support. Participants’ agreement with the statements was significantly associated with the type of support (*F*(2, 74) = 9.03, *P* < 0.001, *ηp*^2^ = 0.11). *Post hoc* tests show that there were significantly higher scores for Arena support than for support from family and friends not at the Arena (*P* = <0.001) and support from one's workplace (*P* = <0.001). However, there was no difference between scores for support from family and friends not at the Arena and support from one's workplace (*P* = 1.00).

To test that the pattern of results that emerged from the analysis was not a result of differences in how the items were worded, the same analysis was carried out using just the phrase, ‘showed a lot of understanding of what I've been through’, which was common to three questions in the survey (questions 79, 89 and 90). This procedure produced the same significant overall difference as for the full scales (*F*(2, 73) = 9.29, *P* < 0.001, *ηp*^2^ = 0.11), and the same pattern in the *post hoc* tests.

##### Longitudinal Hub scores and social support

We compared the mean scores of the Hub respondents categorised as mild, moderate and severe at 3- or 6-month and 30- or 36-month assessment on the five social cure variables. One-way ANOVA showed that there were significant differences between the response groups on two of the support factors; family and friends not at the Arena and efficacy. *Post hoc* tests (Tukey's honestly significant difference) showed that there were significantly lower scores in the severe group as compared with the other groups at both initial and final assessment time points (Supplementary Tables 10 and 11), implying that lower scores on these variables may be associated with chronic trajectories of distress.

#### Correlations

Table 6 shows the associations of the social cure variables with post-event distress, secondary stressors and current mental well-being (WEMWBS score).

**Table 6:** Correlations between social cure variables, Warwick–Edinburgh Mental Wellbeing Scale scores, post-event distress and secondary stressors

	Pearson *r* (*P*-values)						
	Efficacy	Arena support	Support from family and friends not at the Arena	Support from one's workplace	WEMWBS	Post-event distress	Secondary stressors
Social identity (*N* = 84)	0.416 (<0.001***)	0.586 (<0.001***)	0.099 (0.369)	−0.029 (0.805)	0.273 (0.012*)	−0.139 (0.207)	0.016 (0.887)
Efficacy (*N* = 84)		0.273 (0.012*)	0.319 (0.003**)	0.211 (0.071)	0.510 (<0.001***)	−0.250 (0.022*)	−0.285 (0.009**)
Arena support (*N* = 84)			0.200 (0.068)	−0.004 (0.974)	0.208 (0.057)	−0.069 (0.535)	0.021 (0.851)
Support from family and friends not at the Arena (*N* = 84)				0.083 (0.481)	0.338 (0.002**)	−0.242 (0.027*)	−0.226 (0.038*)
Support from workplace (*N* = 74)					0.157 (0.181)	0.185 (0.115)	0.007 (0.952)
WEMWBS (*N* = 84)						−0.170 (0.123)	−0.283 (0.009**)
Post-event distress (*N* = 84)							0.740 (<0.001***)

WEMWBS, Warwick–Edinburgh Mental Wellbeing Scale.

*P<0.05, **P<0.01, ***P<0.001.

Current mental health was most associated with efficacy (large effect), social identity and support from family and friends not at the Arena (moderate effects). As expected, social identity was associated with Arena support, efficacy and post-event distress (negatively). Efficacy was associated with Arena support, support from family and friends not at the Arena, post-event distress (negatively) and secondary stressors (negatively). Also, as expected, support from family and friends not at the Arena was negatively associated with post-event distress and secondary stressors. Arena support was not associated with post-event distress or current mental well-being. Secondary stressors and support from one's workplace were not associated with any other measures.

##### Mediation models

Results are reported for the two proposed mediation model structures (models 1 and 2), controlling for age and secondary stressors. For each model, we report the indirect effects.

For model 1, we used PROCESS version 3.3 (Model 6) for Windows (Haskayne School of Business, University of Calgary, Canada; see https://www.processmacro.org/download.html) to test the pathway from social identity to current mental well-being via Arena support and then efficacy.^41^ Results based on 10 000 bootstrapped samples indicated no indirect effect (*b* = 0.19, 95% bias-corrected and accelerated confidence intervals (BCa CI) −0.742 to 0.851). Age was a non-significant covariate (*b* = −0.03, *P* = 0.74), and secondary stressors were a significant covariate (*b* = −2.95, *P* = 0.02).

For model 2, using the same analysis, we tested the pathway from Arena support to current mental well-being via social identity and then efficacy (see Fig. 1). Figure 1 shows a non-significant pathway between Arena support and efficacy, and this is the key difference between the two models. Results based on 10 000 bootstrapped samples indicated that there was a significant indirect effect (*b* = 0.15, 95% BCa CI 0.283–3.327). Therefore, this is the preferred model. Secondary stressors were a significant covariate (*b* = −2.99, *P* = 0.02). Age was, again, a non-significant covariate (*b* = −0.05, *P* = 0.52).

Both models were considered to be just identified, meaning the fit is considered perfect to the observed correlation matrix, and so goodness-of-fit measures (that are compared to the saturated model) were not appropriate.

## Discussion

### The sample

Our sample is of predominantly female participants. That might appear as a bias, but it was representative of the gender imbalance of the people who attended the Arena event. Therefore, their experiences may reflect the much larger population of people affected by the incident. However, our findings with this female dominated sample may not be typical of other events with more diverse populations.

### Experiences of distress

Consistent with our earlier interview study,^13^ excessive arousal and vigilance at social gatherings and in public places, fear of recurrence of the event and upsetting thoughts or images of the event were the most reported experiences of distress in this larger survey in the first 3 months following the incident. In our earlier study, we also found evidence suggesting that certain initial psychosocial responses, such as social withdrawal and changes in mood, were associated with more severe and enduring distress. This association has also been reported recently in relation to terrorist attacks^42–44^ and natural hazards,^45^ suggesting that certain early experiences might serve as markers of the risk of longer-term distress.

We found that specific post-event responses, such as social withdrawal, irritability and memory difficulties, were the items most associated with lower WEMWBS scores. Based on our exploratory factor analysis, social withdrawal/physical symptoms appears to be a genuine latent construct of post-event distress in our sample. However, regression analysis showed that the social withdrawal/physical symptoms factor was not predictive of mental well-being 3 years post-event. Impaired everyday functioning and change in affect were predictive of current well-being, with moderate effect sizes. However, they only accounted for a small amount of the variance in post-event distress. Therefore, it is unclear whether they represent legitimate constructs of post-event distress with any predictive value.

### Trajectories of distress and recovery

Analysis of the cross-sectional survey and longitudinal Hub data has provided notable findings on the course and patterns of distress in our cohort of survivors. Comparison with WEMWBS population norms showed that the mental well-being of our sample was significantly lower than the general population. The event continued to have an impact on survivors’ mental well-being more than 3 years later. Our participants had a mean score more than 9 points below the UK national average; differences of 3 points or more are viewed as clinically important in outcome studies.^46^ Studies comparing WEMWBS scores with a validated measure of depression indicate that 42% of our sample of survivors could be at ‘high risk of major depression’, and 60% should be considered in ‘high risk of psychological distress and increased risk of depression’.^47^

Longitudinal analysis of the Hub data also provides evidence of enduring impairment. There was a significant fall in the GAD-7 and TSQ mean scores, but no significant changes in the PHQ-9 or WSAS scores over 3 years. Scores in the severe group did not reduce significantly over time on any of the Hub measures. In addition, 69% of people with a moderate or severe initial reaction were still categorised as moderate or severe on Hub metrics 3 years after the Arena attack. Thus, slow recovery and chronicity are common trajectories for the moderate and severe response groups in our sample.

These findings are consistent with the typical broad pattern of psychosocial responses or trajectories reported elsewhere in the literature,^48–50^ and provide evidence that people's responses to major incidents fall into three main groups (Supplementary Material): short-term distress, more persistent distress and slower recovery, and high stress and deteriorating responses.

Previous work on the impact of major incidents indicates that the intensity of initial distress is strongly associated with enduring and debilitating distress.^44^ Some studies suggest that distress reaches a peak in the year following the event, and then slowly improves with recovery, ranging from several months to 2 years.^51,52^ However, a number of long-term studies report persistent distress and enduring psychosocial repercussions for some people over many years.^22,53^ Many of our survivors who experienced mild and moderate responses were still experiencing some degree of distress and impaired functioning 3 years after the event. Thus, the existing literature might underestimate the number of people who take a long time to recover.

### The impact of secondary stressors

Secondary stressors have the potential to exacerbate distress during and following major incidents.^14,15^ However there is a dearth of research identifying which secondary stressors are particularly associated with mass terrorist events, and how they can be targeted by more effective and timely psychosocial interventions. Our findings advance understanding of secondary stressors and elucidate the stressors that affected coping and recovering in our respondents.

Our analysis showed that disruption to relationships with family and friends has a significant association with our survivors’ current mental well-being. This amplifies the findings from our earlier interview study.^13^ We speculate that the quality of relationships may be a salient influence on coping and rate of recovery following terrorist attacks. We suggest that enquiring about the quality of relationships should be central in assessing risk and need.

The longitudinal Hub data also showed that there may be evidence of a linear relationship between secondary stressors and longer-term outcome, which suggests that secondary stressors might be associated with the enduring risk of mental health disorders. However, we note that all of the factors arising from analysis of secondary stressors were significantly correlated with post-event distress (Supplementary Table 12). This could indicate a bidirectional relationship between post-event distress and secondary stressors, and complicates any simple conclusions about a causative interpretation of relationship between secondary stressors and longer-term outcomes.

### Social cure processes

In line with previous research, our participants showed high levels of identification with others who had been at the Arena.^27^ We found that our participants showed greater appreciation, or perceived greater effects, of support from other people who had been at the Arena than support from work colleagues, or family and friends who had not been at the Arena. This finding corroborates our interview study finding that people who share the common experience of a disaster are perceived as more able to understand participants’ feelings, and participants feel more able to disclose to them.^27^

Only the social support variable concerning family members and friends not at the Arena was shown to relate directly to current mental well-being and post-event distress, suggesting that support from families might reduce distress both immediately and in the longer term.

We tested two social cure models and found that there was greater statistical support for a model in which perceived support from other people present at the Arena leads to efficacy and current mental health via shared identity^28^ (compared with Bokszczanin^54^) than a model in which shared identity leads to efficacy and current mental health via social support.^20^ Perhaps, in the context of a recovery period extended over several years, experiencing support from others at the Arena was crucial to people forming and sustaining an identity defined by the event. Typically, disaster communities and their associated identities are short lived.^16,55^ Conscious action is needed to keep them alive and sustain their benefits.^56^

Although we expected support from other people at the Arena to be directly associated with reduced distress, we did not find that here. However, we did find a relationship of support from others at the Arena with social identity and efficacy. In turn, efficacy was strongly negatively associated with post-event distress, chronic trajectories of distress on the Hub metrics and current mental well-being, in line with previous research.^29^ Importantly, as our qualitative findings suggest,^27^ it seems that the beneficial effects of identifying with, and receiving support from, other people at the Arena is indirect, with each affecting beliefs about one's abilities to cope, which had a direct effect on current mental well-being.

### Measurement issues

#### Hub questionnaires

Participants’ initial TSQ scores did not correlate with their final distress categories. This suggests that the TSQ was the weakest predictor of long-term recovery trajectory of all of the Hub metrics, and is consistent with evidence in the literature of the low specificity and sensitivity of the TSQ.^57^ The WSAS and PHQ-9 were better predictors of mental well-being at 3 years, compared with the TSQ and GAD-7.

#### The WEMWBS

There were very high correlations between the final (30 or 36 month) Hub metrics and WEMWBS scores. WEMWBS was not designed as an instrument to detect mental illness, but very low scores may indicate need for clinical examination. The WEMWBS can measure a wider range of distress and everyday functioning than metrics from other measures that are intended to detect psychopathology. This suggests that it may be appropriate to utilise a measure of mental well-being, such as the WEMWBS, in early assessment and intervention programmes after major incidents.

### Limitations and strengths

Our study is an exploratory analysis. We acknowledge its limitations and recommend caution when interpreting the results.

Sample size and bias may limit the generalisability of our findings and the power of the results reported herein, particularly in the small group comparisons. Comparison of initial assessment data for all Hub registrants completing 3 and/or 6 month assessments (*N* = 1880) with our survey responders showed that there were statistically significant differences between the two groups on two of the Hub's measures (the PHQ-9 and WSAS), with more people categorised with severe initial reactions in our survey group. The age profile of our survey respondents was skewed toward older age compared with all Hub registrants. Our respondents were recruited from a subset of 262 survivors who registered with the Hub in the aftermath of the Arena attack and had expressed an interest in participating in research. We cannot assert that our respondents’ experiences are representative of all survivors of the event. We think that the way in which we recruited our sample made it more likely that fewer of our participants experienced short-term distress, because our sample was composed of people whose distress was of sufficient duration and/or severity to take them to the Hub.

The items in our online survey, except the WEMWBS questionnaire and the social identity measures, were not previously validated. This might have affected the quality of the data in terms of comparability and credibility. The self-report and retrospective nature of our survey data collection also brings limitations, in that the data are not supported by clinical interviews to validate our respondents’ experiences of distress, secondary stressors or social support.

A strength of our study is that we were able to draw on longitudinal data, collected at regular intervals over 3 years, and combine it with the self-report data to mitigate the potential biases. An asset of our online survey is that much of its content was based on learning from participants’ experiences and opinions gained from an earlier qualitative analysis of semi-structured interviews with a purposive sample of survivors of the Arena attack. The qualitative study in the first phase informed and complements the quantitative study reported here. A benefit of this co-productive, mixed methodology is that it lends itself to more context-specific analysis of the impact of major incidents. It facilitates designing bespoke psychosocial interventions tailored to the specific risks and needs of various incidents, as opposed to a ‘one-size-fits-all’ approach.

Previously, we outlined three approaches used to define distress: epidemiological, typological and experiential. Our approach to understanding distress has straddled these differing perspectives by drawing on a validated measure of current mental well-being (WEMWBS score), the longitudinal clinical data from the Hub and our participants’ subjective reports of their post-event distress. We believe that these complementary viewpoints generate congruent findings in relation to distress trajectories, secondary stressors and social cure processes.

### Practical implications

#### The importance of psychosocial care

Distress was extremely common and enduring for most of our participants, including people who were ‘mildly’ affected, but they did not reach the threshold for mental health disorders or specialist care. They represent a large group of people whose suffering warranted validation, and many of them desired access to psychosocial care. This requires agencies to act together to broaden the scope of approaches to recovery to include monitoring distress, associated experiences and effects on functioning. Clinical interviews are important in deciding the severity of people's responses and the nature of interventions offered.

#### Assessing and monitoring people in need

Measures identifying symptoms of mental disorders have inherent limitations in specificity and sensitivity, and they can lead to inappropriate, premature pathways to specialist mental healthcare for some people or overlook the large group of people who are distressed and may require psychosocial care. Our view is that overreliance on instruments that measure people's distress from one perspective (e.g. of symptoms of mental disorder) has inherent limitations. Our study combined wider perspectives on distress with measures of disorder, measures of broader categories of functioning and subjective accounts of the experience of distress. Our experience highlights the importance of considering all perspectives when assessing survivors’ needs for psychosocial care following major incidents.

We advocate brief, narrative assessment and regular monitoring to identify people who may need more personalised psychosocial care in the early stages. This could be supplemented by psychosocial tools that assess mental well-being, overall functioning and coping abilities; the support available within each person's social context and, particularly, their closest relationships; and any hindrances to accessing social support.^58–61^

#### Strengthening the contributions of families, friends and significant others

People need acknowledgement, and emotional and practical support from their close families, friends and colleagues. However, we cannot assume that this happens naturally. SIRE has shown that although family and friends are very important sources of support, their attempts at support can also be unhelpful.^27^ Poor-quality support can exacerbate distress and prolong recovery. Hence services should focus on helping to mobilise support from each person's network, and offer psychosocial interventions with families, to enable members to seek social support and validation, facilitate intrafamilial communication processes and increase families’ understanding of survivors’ experiences.

Activities that can have positive effects include bringing together people with shared experiences. They can be facilitated by enabling survivors to contact other people who have been affected or have had similar experiences. These contacts can also be organised in self-help groups. Peer support offered by people who have shared experiences provides mutual support and can be a catalyst for social validation, information exchange and develop social identification. Outreach services can also facilitate workshops and visits to create spaces wherein people can talk about their experiences and come together, and these formal group meetings can lead to informal meetings.

#### Attending to the impact of secondary stressors

Although exposure to primary stressors may increase distress in the short term, the long-term course of distress may depend on persistent exposure to secondary stressors. They are tractable and recognising them should be included in psychosocial care programmes to mitigate the long-term course of distress and restore functioning. Thus, the social model of secondary stressors enables a holistic approach to conceptualising and intervening to remedy many of the longer-term and widespread negative psychosocial effects of major incidents.^5^^,14^

In conclusion, our exploratory study raises issues that require further debate. We recommend longitudinal research, using larger sample sizes, to confirm or refute our findings. Nonetheless, our main conclusions are that distress is enduring for many people, even those with relatively ‘mild’ impairments who do not need formal assessment and specialist healthcare. Also, secondary stressors are associated with enduring distress and possible mental health disorder, and should be central in assessing risk and need following major incidents. The quality of close relationships is pivotal to long-term outcome. Finally, constructive support from families, friends and people with shared experiences at the event are key to social cure processes that promote recovery. Planners and practitioners should take these processes into account when they design services for future incidents.

## Supporting information

Stancombe et al. supplementary material Stancombe et al. supplementary material

## Data Availability

The authors confirm that the data supporting the findings of this study are available within the article and the supplementary materials or from the corresponding author, J.S., upon reasonable request.
