# Supplementary material for: Trajectories of distress and recovery, secondary stressors and social cure processes in people who used the resilience hub after the Manchester Arena bombing
Source: BJPsych Open. 2023 Aug 8;9(5):e143. doi: 10.1192/bjo.2023.527 (PMC10594089; doi:10.1192/bjo.2023.527)
Supplement: Stancombe et al. supplementary material [file S2056472423005276sup001.docx]

**TRAJECTORIES OF DISTRESS AND RECOVERY, SECONDARY STRESSORS, AND SOCIAL CURE PROCESSES IN PEOPLE WHO USED THE RESILIENCE HUB AFTER THE MANCHESTER ARENA BOMBING**

**SUPPLEMENTARY MATERIALS**

**This file consists of four annexes that are supplementary to the main paper.**

**ANNEX A: SIRE ONLINE SURVEY**

**MANCHESTER RESILIENCE HUB: SOCIAL INFLUENCES ON RECOVERY ENQUIRY (SIRE)**

**Introduction**

The Manchester bombing was very distressing for many people who attended the Arena that night. We're interested in how you've been coping since the event and what you have found helpful in recovering after the event. We're particularly interested in hearing about the support you might have received from friends, families, and other groups as well as things that might have added to your stress or delayed your recovery. Learning about your experience will help us provide more timely and effective services in future.

**Section 1: Some Information About You Please**

First there are some questions about you. We are collecting this information, so we know something about the people who volunteer for the research. We will not publish this information in a way that identifies you. Please note that all information collected is treated in a confidential manner and will only be used to produce statistical information.

| **Disability** |  |
| --- | --- |
| Do you consider yourself to be a disabled person | YES / NO |
| If you answered yes, please tick the boxes below that apply to you | Please tick any of the descriptions below that apply to you |
| Profound to mild deafness |  |
| Speech impairment |  |
| Blind or partially sighted |  |
| Mobility or physical impairment – limits or restricts physical movement, coordination, or manual dexterity |  |
| Long-standing illness or diagnosed health condition e.g., cancer, HIV, diabetes, chronic heart disease, rheumatoid arthritis |  |
| Learning or developmental disability e.g., Down Syndrome, autism, or dyslexia |  |
| Mental ill health e.g., bi-polar disorder, schizophrenia, depression |  |
| Impaired memory/concentration or ability to understand e.g., stroke, dementia, dyslexia, head injury |  |
| Other – please state | Free text |

**My Age**

Please insert your age in complete years on the date you fill in this survey

If you would prefer not to say, please tick this box

| **My Gender** | Please tick |
| --- | --- |
| Female |  |
| Male |  |
| Do not wish to declare |  |

| **My Relationship Status** | Please tick |
| --- | --- |
| Marriage |  |
| Civil Partnership |  |
| Cohabiting |  |
| Single |  |
| Other |  |
| Prefer not to say |  |

| **My Ethnicity** | Please tick |
| --- | --- |
| Arab |  |
| Asian/British Bangladeshi |  |
| Asian/British Indian |  |
| Asian/British Other |  |
| Asian/British Pakistani |  |
| Black/British African |  |
| Black/British Caribbean |  |
| Black/British Other |  |
| Chinese |  |
| Mixed Other |  |
| Mixed White/Asian |  |
| Mixed White/Black African |  |
| Mixed White/Black |  |
| White British |  |
| White Irish |  |
| White Other |  |
| Other |  |
| Prefer not to say |  |

| **My Sexual Orientation** | Please tick |
| --- | --- |
| Bisexual |  |
| Gay |  |
| Heterosexual |  |
| Lesbian |  |
| Prefer not to say |  |

| **Faith Religion or Beliefs** | Very Strongly Disagree |  |  |  |  | Very Strongly Agree |
| --- | --- | --- | --- | --- | --- | --- |
| Faith or religious beliefs are extremely important to me | ❑ | ❑ | ❑ | ❑ | ❑ | ❑ |

| **Residence and Occupation** | |
| --- | --- |
| In which town or city do you live? | Free text |
| My occupation at the time of the Event was | Free text |

| **My Household Income at the Time of the Event in 2017 was:** | Please tick the appropriate income |
| --- | --- |
| £0 to £10,000 |  |
| £10,001 to £20,000 |  |
| £20,001 to £30,000 |  |
| £30,001 to £50,000 |  |
| £50,001 to £70,000 |  |
| £70,001 to £100,000 |  |
| More than £100,001 |  |
| Prefer not to say |  |

| **My experiences of stress and distress BEFORE the Event in 2017 were:** | **Please tick the box that best describes you** |
| --- | --- |
| I coped with stress without becoming too distressed or anxious |  |
| I had a tendency to become distressed or anxious when facing stressful situations |  |
| I had one or more episodes of emotional or psychological difficulties which significantly affected my everyday function |  |
| I had one or more episodes of emotional or psychological difficulties after a serious adverse event that significantly affected my everyday function |  |

**Section 2: Your Experiences in the First 3 Months after the Arena Event**

The statements below are about people’s experiences of distress following the Arena event.

Please tick the box that best describes your experience of each in the FIRST 3 MONTHS after the Event.

| **STATEMENTS** | Not at all | Rarely | Some of the time | Often | All of the time |
| --- | --- | --- | --- | --- | --- |
| I felt numb | 1 | 2 | 3 | 4 | 5 |
| I felt shocked | 1 | 2 | 3 | 4 | 5 |
| I felt intensely fearful or very anxious | 1 | 2 | 3 | 4 | 5 |
| I felt helpless or hopeless about myself, other people or the world | 1 | 2 | 3 | 4 | 5 |
| I was very worried about the risk of another terrorist attack | 1 | 2 | 3 | 4 | 5 |
| I felt guilt, ashamed or in some way to blame | 1 | 2 | 3 | 4 | 5 |
| I had angry outbursts | 1 | 2 | 3 | 4 | 5 |
| I could not feel pleasure in activities that were usually pleasurable for me | 1 | 2 | 3 | 4 | 5 |
| I had problems remembering things | 1 | 2 | 3 | 4 | 5 |
| I had difficulties concentrating on things | 1 | 2 | 3 | 4 | 5 |
| I felt confused | 1 | 2 | 3 | 4 | 5f |
| I was unsure about where I was or about what was the date | 1 | 2 | 3 | 4 | 5 |
| I had upsetting thoughts or images in my mind about the event | 1 | 2 | 3 | 4 | 5 |
| I felt distant or cut off from people around me | 1 | 2 | 3 | 4 | 5 |
| I tried not to remember, talk about or have feelings about the event | 1 | 2 | 3 | 4 | 5 |
| I had lower confidence in myself than usual | 1 | 2 | 3 | 4 | 5 |
| I was unusually alert or on my guard | 1 | 2 | 3 | 4 | 5 |
| I lost skills that I had before the event | 1 | 2 | 3 | 4 | 5 |
| I avoided people and activities that I would usually enjoy | 1 | 2 | 3 | 4 | 5 |
| I wanted to be by myself most of the time | 1 | 2 | 3 | 4 | 5 |
| I was irritable without good reason and took it out on other people or things | 1 | 2 | 3 | 4 | 5 |
| I had [serious](https://www.collinsdictionary.com/dictionary/english/serious) [disagreement](https://www.collinsdictionary.com/dictionary/english/disagreement)s or [argument](https://www.collinsdictionary.com/dictionary/english/argument)s with other people that were unusual for me | 1 | 2 | 3 | 4 | 5 |
| I stayed away from activities, people, places or things that reminded me of the event | 1 | 2 | 3 | 4 | 5 |
| I had trouble falling asleep or staying asleep | 1 | 2 | 3 | 4 | 5 |
| I was jumpy or easily startled | 1 | 2 | 3 | 4 | 5 |
| I had headaches | 1 | 2 | 3 | 4 | 5 |
| I had persistent physical symptoms that I did not have before the event | 1 | 2 | 3 | 4 | 5 |
| I had reduced appetite | 1 | 2 | 3 | 4 | 5 |
| My energy levels were lower than before the event | 1 | 2 | 3 | 4 | 5 |

**Section 3: Your Mental Wellbeing in the Last 4 Weeks: The 14 item Warwick–Edinburgh Mental Well-being Scale**

The statements below are about your current feelings and thoughts.

Please tick the box that best describes your experience of each over THE LAST 4 WEEKS

|  | None of the time | Rarely | Some of the time | Often | All of the time |
| --- | --- | --- | --- | --- | --- |
| I’ve been feeling optimistic about the future | 1 | 2 | 3 | 4 | 5 |
| I’ve been feeling useful | 1 | 2 | 3 | 4 | 5 |
| I’ve been feeling relaxed | 1 | 2 | 3 | 4 | 5 |
| I’ve been feeling interested in other people | 1 | 2 | 3 | 4 | 5 |
| I’ve had energy to spare | 1 | 2 | 3 | 4 | 5 |
| I’ve been dealing with problems well | 1 | 2 | 3 | 4 | 5 |
| I’ve been thinking clearly | 1 | 2 | 3 | 4 | 5 |
| I’ve been feeling good about myself | 1 | 2 | 3 | 4 | 5 |
| I’ve been feeling close to other people | 1 | 2 | 3 | 4 | 5 |
| I’ve been feeling confident | 1 | 2 | 3 | 4 | 5 |
| I’ve been able to make up my own mind about things | 1 | 2 | 3 | 4 | 5 |
| I’ve been feeling loved | 1 | 2 | 3 | 4 | 5 |
| I’ve been interested in new things | 1 | 2 | 3 | 4 | 5 |
| I’ve been feeling cheerful | 1 | 2 | 3 | 4 | 5 |

**Section 4: Things That Cause You to Feel Stressed**

Listed below are a number of difficult or stressful things that sometimes happen to people after major events.

Please rate how frequently since the Event you have experienced stress with:

Rated on a Likert 5-point frequency scale (1 Never 2 rarely 3 occasionally 4 moderate amount 5 A great deal)

| 1 | Loss of income |
| --- | --- |
| 2 | Loss or lack of employment |
| 3 | New or additional pressures on me at work |
| 4 | Difficulties with making an application to the compensation scheme |
| 5 | Difficulties with the unfairness of the compensation scheme |
| 6 | New or continuing physical health problems |
| 7 | New or continuing mental health problems |
| 8 | Lack of access to the physical healthcare I need |
| 9 | Lack of access to the mental healthcare I need |
| 10 | Difficulties with finding information and support |
| 11 | Lack of education opportunities or facilities |
| 12 | Exposure to negative reports in the news media |
| 13 | Social media |
| 14 | The response from the community |
| 15 | Disruption to relationships with my friends |
| 16 | Disruption to relationships within my family |
| 17 | Disruption to my leisure and relaxation activities |
| 18 | Spending more time helping my family, friends or other people in the community |

**Section 5: Your Experiences Since the Event**

**Part A**

Here are some activities that we would like you to think about. Please think back over the years and months since the Arena Event.

Please rate the extent to which other people have shown the following towards you in response to the Manchester Arena Bombing in May 2017

|  | | Not at all |  |  |  |  |  | To a very great extent |
| --- | --- | --- | --- | --- | --- | --- | --- | --- |
| 1 | They gave me emotional support | ❑ | ❑ | ❑ | ❑ | ❑ | ❑ | ❑ |
| 2 | They showed respect for me | ❑ | ❑ | ❑ | ❑ | ❑ | ❑ | ❑ |
| 3 | They showed concern for my needs | ❑ | ❑ | ❑ | ❑ | ❑ | ❑ | ❑ |

Have you been employed since the Arena Event? Yes / No

If you have been employed since the Arena Event, please answer the following three questions:

|  | | Not at all |  |  |  |  |  | To a very great extent |
| --- | --- | --- | --- | --- | --- | --- | --- | --- |
| 4 | People in my workplace showed a lot of understanding of what I’ve been through | ❑ | ❑ | ❑ | ❑ | ❑ | ❑ | ❑ |
| 5 | The care and support of my colleagues at work has helped me | ❑ | ❑ | ❑ | ❑ | ❑ | ❑ | ❑ |
| 6 | The care and support of my employers has helped me | ❑ | ❑ | ❑ | ❑ | ❑ | ❑ | ❑ |

**Part B**

The next set of statements below describe some people’s experiences while recovering following the Arena event.

Please answer all questions by ticking the box that best describes your opinions since the Event.

|  | | Very Strongly Disagree |  |  |  |  |  | Very Strongly Agree |
| --- | --- | --- | --- | --- | --- | --- | --- | --- |
| 1 | I’ve found it easy to talk about my experiences with my family and friends who were NOT at the Arena | ❑ | ❑ | ❑ | ❑ | ❑ | ❑ | ❑ |
| 2 | I’ve found it easy to talk about my experiences with my family and friends who were at the Arena | ❑ | ❑ | ❑ | ❑ | ❑ | ❑ | ❑ |
| 3 | I’ve found it easy to talk about my experiences with other people who were at the Arena | ❑ | ❑ | ❑ | ❑ | ❑ | ❑ | ❑ |
| 4 | It was helpful to share my feelings with my family and friends who were at the Arena | ❑ | ❑ | ❑ | ❑ | ❑ | ❑ | ❑ |
| 5 | It was helpful to share my feelings with other people who were at the Arena | ❑ | ❑ | ❑ | ❑ | ❑ | ❑ | ❑ |
| 6 | It was helpful to share my feelings with family and friends who were not at the Arena | ❑ | ❑ | ❑ | ❑ | ❑ | ❑ | ❑ |
| 7 | My family and friends who were at the concert showed a lot of understanding of what I’ve been through | ❑ | ❑ | ❑ | ❑ | ❑ | ❑ | ❑ |
| 8 | My family and friends who did not go to the concert showed a lot of understanding of what I’ve been through | ❑ | ❑ | ❑ | ❑ | ❑ | ❑ | ❑ |
| 9 | Other people at the concert showed a lot of understanding of what I’ve been through | ❑ | ❑ | ❑ | ❑ | ❑ | ❑ | ❑ |
| 10 | The care and support of my family has helped me | ❑ | ❑ | ❑ | ❑ | ❑ | ❑ | ❑ |
| 11 | The care and support of my friends has helped me | ❑ | ❑ | ❑ | ❑ | ❑ | ❑ | ❑ |
| 12 | The care and support of others who were at the concert has helped me | ❑ | ❑ | ❑ | ❑ | ❑ | ❑ | ❑ |
| 13 | it was helpful to make contact with other people who had been at the concert via social media | ❑ | ❑ | ❑ | ❑ | ❑ | ❑ | ❑ |
| 14 | It was helpful to meet face-to-face with other people who had been at the event | ❑ | ❑ | ❑ | ❑ | ❑ | ❑ | ❑ |
| 15 | It was helpful to have reassurance from professionals that my reaction to the event was common and expected | ❑ | ❑ | ❑ | ❑ | ❑ | ❑ | ❑ |
| 16 | Together with other people affected by the event, I feel able to change my situation | ❑ | ❑ | ❑ | ❑ | ❑ | ❑ | ❑ |
| 17 | Those of us affected by the event feel in control of things | ❑ | ❑ | ❑ | ❑ | ❑ | ❑ | ❑ |
| 18 | I feel capable of accessing the services I need | ❑ | ❑ | ❑ | ❑ | ❑ | ❑ | ❑ |
| 19 | I feel able to organise things to meet my needs | ❑ | ❑ | ❑ | ❑ | ❑ | ❑ | ❑ |
| 20 | I feel a bond with people who shared the same experience as me at the Arena | ❑ | ❑ | ❑ | ❑ | ❑ | ❑ | ❑ |
| 21 | I feel solidarity with people who shared the same experience as me at the Arena | ❑ | ❑ | ❑ | ❑ | ❑ | ❑ | ❑ |
| 22 | I identify with people who shared the same experience as me at the Arena | ❑ | ❑ | ❑ | ❑ | ❑ | ❑ | ❑ |
| 23 | Looking back, were there any other sources of support that you have received that have been helpful? | Free text | | | | | | |
| 24 | Looking back, is there any help or support that you would have liked to have been given after the Event? | Free text | | | | | | |

THANK YOU FOR PARTICIPATING IN THIS RESEARCH STUDY AND COMPLETING THE SURVEY

**ANNEX B: ITEMS IN THE SURVEY NOT INCLUDED IN THE ANALYSIS REPORTED IN THE PAPER**

We did not include the single item measuring experiences of stress and distress before the event in the analyses presented here. We also included items for parents and carers on the impact of the events on their child or children plus three free-text questions on the same topic. They are not included as part of the analysis. In addition, two of the ‘support’ items were not included in the three variables constructed and used in the analysis. We also included an item measuring efficacy in a different domain (accessing services), but we do not include that in the present analysis.

**ANNEX C: PEOPLE’S RESPONSES TO MAJOR INCIDENTS**

The ways in which people respond to emergencies and disasters fall into three main groups:

1. Short-term distress (around 50% of people) in the immediate aftermath. Most people in this group are able to function satisfactorily in the short and medium term although they may show temporary dysfunction while they are distressed.
2. More persistent distress and slower recovery (around 30%) that may be of longer duration than the experiences of people in the first group. This group represents a substantial number of people, who can experience continuing distress and, sometimes dysfunctional problems after major incidents without reaching the threshold criteria for referral to mental healthcare services. People in this group require assessment and psychosocial care. At present, there is an absence of validated formal psychosocial interventions for people in this group
3. High stress and deteriorating responses (around 20%) of people who may develop a mental disorder in the short-, medium-, or longer-terms and may require specialist assessment and mental healthcare.

The first two groups relate to people with different levels of psychosocial need, while the third is composed of people who develop mental disorders indicating their possible requirement for biomedical mental healthcare interventions. Importantly, everybody is likely to benefit from social support and it may act in a preventative capacity.

**ANNEX D: SUPPLEMENTARY TABLES CITED IN THE ANALYSIS REPORTED IN THE PAPER**

**Table 1S: Demographic Details of Participants**

| **Demographic Categories** | | **N (84)** | **%** |
| --- | --- | --- | --- |
| **Gender** | Female  Male  Prefer not to say and not known | 71  10  3 | 85  12  3 |
| **Age** (Mean 37.39; SD 13.56) | 18 to 20  21 to 40  41 to 50  51 plus  Not known | 2  40  21  20  1 | 2  47  25  23  1 |
| **Ethnicity** | White  Asian/British Pakistani/Other  Prefer not to say or Not known | 79  2  3 | 94  2  3 |
| **Sexuality** | Heterosexual  Gay  Lesbian  Bisexual  Prefer not to say or Not Known | 67  5  2  5  5 | 80  6  2  6  6 |
| **Relationship** | Single  Married/Cohabiting/Civil partnership  Other  Prefer not to say or Not known | 36  36  9  5 | 42  41  11  6 |
| **Faith important** | Strongly agree and Agree  Neither agree or Disagree  Disagree and Strongly disagree  Not known | 13  38  31  2 | 16  45  37  2 |
| **Disability** | Yes (Mental health, Physical, Diagnosed health condition, Learning)  No  Not known | 10  73  1 | 12  87  1 |
| **Household income** | £0 to £20,000  £20,000 to £50,000  £50,000 plus  Prefer not to say or Not known | 21  28  18  17 | 25  27  22  20 |
| **Stress (Your experience of distress before the Arena bombing)** | Coped with stress  Some anxiety  Previous psychological difficulties  Not known | 52  21  9  2 | 62  25  11  2 |
| **Location** | Greater Manchester  Other North West  North East, Yorkshire, Humberside  Midlands  South West  Scotland  Wales  Not known | 18  22  25  7  3  5  3  1 | 21  26  29  8  4  6  4  1 |
| **Occupation** | Managers, Professional & Technical Occupations  Administrative and secretarial occupations  Skilled trades occupations  Caring, leisure and other service occupations  Sales and customer service occupations  Elementary occupations  Employed unspecified  Student  Retired, Unemployed or Not known | 23  14  1  10  7  4  1  18  6 | 28  17  1  12  8  5  1  21  7 |

**Table 2S: Mean Differences of Post-Event Distress Items and the Full-scale Scores**

| **Post-event Distress Questions** | | **N** | **Mean** | **SD** | **T**  **Test** | **P** | **Mean difference (from scale mean 3.40)** | **95% CI** | | **Post-event Distress Factor** |
| --- | --- | --- | --- | --- | --- | --- | --- | --- | --- | --- |
|  |  |  |  |  |  |  |  | **Lower** | **Upper** |  |
| Q14 | I felt numb | 83 | 3.95 | 0.96 | 5.23 | <0.001* | 0.55 | 0.34 | 0.76 | 4 |
| Q15 | I felt shocked | 84 | 4.17 | 0.79 | 8.91 | <0.001* | 0.77 | 0.60 | 0.94 | 4 |
| Q16 | I felt intensely fearful or very anxious | 84 | 4.04 | 1.17 | 5.00 | <0.001* | 0.64 | 0.38 | 0.89 | 2 |
| Q17 | I felt helpless or hopeless about myself, other people or the world | 82 | 3.74 | 1.23 | 2.54 | 0.013* | 0.34 | 0.07 | 0.61 | 4 |
| Q18 | I was very worried about the risk of another terrorist attack | 84 | 4.12 | 1.09 | 6.04 | <0.001* | 0.72 | 0.48 | 0.96 | 2 |
| Q19 | I felt guilt, ashamed or in some way to blame | 84 | 3.31 | 1.47 | -0.56 | 0.575 | -0.09 | -0.41 | 0.23 | 5 |
| Q20 | I had angry outbursts | 84 | 2.87 | 1.19 | -4.09 | <0.001* | -0.53 | -0.79 | -0.27 | 5 |
| Q21 | I could not feel pleasure in activities that were usually pleasurable for me | 84 | 3.70 | 1.18 | 2.35 | 0.021* | 0.30 | 0.05 | 0.56 | 4 |
| Q22 | I had problems remembering things | 84 | 3.07 | 1.34 | -2.24 | 0.028* | -0.33 | -0.62 | -0.04 | 3 |
| Q23 | I had difficulties concentrating on things | 84 | 3.70 | 1.27 | 2.18 | 0.032* | 0.30 | 0.03 | 0.58 | 3 |
| Q24 | I felt confused | 84 | 3.20 | 1.28 | -1.42 | 0.160 | -0.20 | -0.47 | 0.08 | 3 |
| Q25 | I was unsure about where I was or about what was the date | 83 | 2.36 | 1.30 | -7.26 | <0.001* | -1.04 | -1.32 | -0.75 | 3 |
| Q26 | I had upsetting thoughts or images in my mind about the event | 84 | 4.12 | 0.91 | 7.24 | <0.001* | 0.72 | 0.52 | 0.92 | 4 |
| Q27 | I felt distant or cut off from people around me | 84 | 3.42 | 1.18 | 0.13 | 0.898 | 0.02 | -0.24 | 0.27 | 4 |
| Q28 | I tried not to remember, talk about or have feelings about the event | 84 | 3.29 | 1.37 | -0.77 | 0.446 | -0.11 | -0.41 | 0.18 | _ |
| Q29 | I had lower confidence in myself than usual | 84 | 3.35 | 1.38 | -0.36 | 0.718 | -0.05 | -0.36 | 0.25 | 3 |
| Q30 | I was unusually alert or on my guard | 84 | 4.35 | 0.83 | 10.46 | <0.001* | 0.95 | 0.77 | 1.13 | 2 |
| Q31 | I lost skills that I had before the event | 84 | 2.40 | 1.42 | -6.45 | <0.001* | -1.00 | -1.30 | -0.69 | 3 |
| Q32 | I avoided people and activities that I would usually enjoy | 84 | 3.60 | 1.16 | 1.54 | 0.128 | 0.20 | -0.06 | 0.45 | 1 |
| Q33 | I wanted to be by myself most of the time | 84 | 3.24 | 1.31 | -1.13 | 0.262 | -0.16 | -0.45 | 0.12 | 1 |
| Q34 | I was irritable without good reason and took it out on other people or things | 83 | 3.04 | 1.29 | -2.57 | 0.012* | -0.36 | -0.65 | -0.08 | 5 |
| Q35 | I had serious disagreements or arguments with other people that were unusual for me | 84 | 2.55 | 1.30 | -6.00 | <0.001* | -0.85 | -1.14 | -0.57 | 5 |
| Q36 | I stayed away from activities, people, places or things that reminded me of the event | 84 | 3.85 | 1.18 | 3.47 | 0.001* | 0.45 | 0.19 | 0.70 | 2 |
| Q37 | I had trouble falling asleep or staying asleep | 84 | 3.70 | 1.30 | 2.14 | 0.036* | 0.30 | 0.02 | 0.58 | 3 |
| Q38 | I was jumpy or easily startled | 84 | 4.06 | 1.14 | 5.28 | <0.001* | 0.66 | 0.41 | 0.91 | 2 |
| Q39 | I had headaches | 83 | 2.87 | 1.31 | -3.69 | <0.001* | -0.53 | -0.82 | -0.25 | 1 |
| Q40 | I had persistent physical symptoms that I did not have before the event | 84 | 2.76 | 1.38 | -4.25 | <0.001* | -0.64 | -0.94 | -0.34 | 1 |
| Q41 | I had reduced appetite | 84 | 2.56 | 1.35 | -5.72 | <0.001* | -0.84 | -1.13 | -0.55 | 3 |
| Q42 | My energy levels were lower than before the event | 84 | 3.33 | 1.43 | -0.43 | 0.671 | -0.07 | -0.38 | 0.24 | 1 |

**Table 3S: Pattern Factor Loadings for Exploratory Factor Analysis of the Post-Event Distress Questions**

We conducted a principal axis factor analysis on the 28 post-event distress items (direct oblimin) to explore the extent to which they made up different factors. The Kaiser-Meyer-Olkin (KMO) measure verified the sampling adequacy for the analysis, KMO=0.897. Initial analysis provided eigenvalues for each factor in the data. Five factors had eigenvalues over Kaiser’s criterion of 1 and together explained 64% of the variance in post-event distress scores. However, the first factor alone explained 51% of the variance and had by far the largest eigenvalue. The scree plot likewise indicated one large factor. The factor loadings of the post-event distress items after rotation are shown in the pattern matrix below. Factor 1 contains 5 items, which appear to measure the construct Social Withdrawal/Physical Symptoms (e.g., ‘I had persistent physical symptoms that I did not have before the event’, ‘I wanted to be by myself most of the time’). Factor 2 contains 5 items that appear to represent Fear of Recurrence (e.g., ‘I was very worried about the risk of another terrorist attack’). Factor 3 contains 7 items that index Impaired Everyday Functioning (e.g., ‘I had problems remembering things’). Factor 4 and 5 each contain 5 items, which appear to measure Changes in Affect (e.g., ‘I felt shocked’) and Intense Feelings (e.g., ‘I had angry outbursts’) respectively.

| **Questionnaire items** | | **Factor 1**  **Social withdrawal/ Physical symptoms** | **Factor 2**  **Fear of recurrence** | **Factor 3**  **Impaired everyday function** | **Factor 4**  **Change in affect** | **Factor 5**  **Intense feelings** |
| --- | --- | --- | --- | --- | --- | --- |
| Q40 | I had persistent physical symptoms that I did not have before the event | 0.619 |  |  |  |  |
| Q33 | I wanted to be by myself most of the time | 0.598 |  |  |  |  |
| Q39 | I had headaches | 0.407 |  |  |  |  |
| Q42 | My energy levels were lower than before the event | 0.393 |  |  |  |  |
| Q32 | I avoided people and activities that I would usually enjoy | 0.365 |  |  |  |  |
| Q18 | I was very worried about the risk of another terrorist attack |  | 0.817 |  |  |  |
| Q38 | I was jumpy or easily startled |  | 0.779 |  |  |  |
| Q30 | I was unusually alert or on my guard |  | 0.690 |  |  |  |
| Q16 | I felt intensely fearful or very anxious |  | 0.680 |  |  |  |
| Q36 | I stayed away from activities, people, places or things that reminded me of the event |  | 0.509 |  |  |  |
| Q22 | I had problems remembering things |  |  | -0.938 |  |  |
| Q23 | I had difficulties concentrating on things |  |  | -0.750 |  |  |
| Q24 | I felt confused |  |  | -0.527 | 0.306 |  |
| Q37 | I had trouble falling asleep or staying asleep |  |  | -0.414 |  |  |
| Q41 | I had reduced appetite |  |  | -0.410 |  |  |
| Q25 | I was unsure about where I was or about what was the date |  |  | -0.384 |  |  |
| Q31 | I lost skills that I had before the event |  |  | -0.320 |  |  |
| Q28 | I tried not to remember, talk about or have feelings about the event |  |  |  |  |  |
| Q15 | I felt shocked |  |  |  | 0.719 |  |
| Q14 | I felt numb |  |  |  | 0.593 |  |
| Q21 | I could not feel pleasure in activities that were usually pleasurable for me |  |  |  | 0.458 |  |
| Q26 | I had upsetting thoughts or images in my mind about the event |  |  |  | 0.445 |  |
| Q17 | I felt helpless or hopeless about myself, other people or the world |  |  |  | 0.390 |  |
| Q27 | I felt distant or cut off from people around me |  |  |  |  |  |
| Q20 | I had angry outbursts |  |  |  |  | 0.934 |
| Q34 | I was irritable without good reason and took it out on other people or things |  |  |  |  | 0.649 |
| Q35 | I had serious disagreements or arguments with other people that were unusual for me |  |  |  |  | 0.572 |
| Q29 | I had lower confidence in myself than usual |  |  |  |  | 0.308 |
| Q19 | I felt guilt, ashamed or in some way to blame |  |  |  |  | 0.304 |
| **Eigen Values** | | **15.2** | **1.65** | **1.25** | **1.17** | **1.00** |
| **% of Variance** | | **51.1** | **4.56** | **3.04** | **2.95** | **2.24** |

Extraction Method: Principal Axis Factoring

Rotation Method: Oblimin with Kaiser Normalisation

*Note*. Loadings < .30 are omitted

**Table 4S: Response Categorisation at Initial screening for Survey Responders v All Hub Registrants**

| **Distress at 3 or 6 months** | **PHQ9 (Chi^2^=6.32, p=0.042)** | | | | **GAD7 (Chi^2^=0.001, p=1.00)** | | | | **TSQ (Chi^2^=5.76, p=0.056)** | | | | **WSAS (Chi^2^=10.76, p=0.005)** | | | |
| --- | --- | --- | --- | --- | --- | --- | --- | --- | --- | --- | --- | --- | --- | --- | --- | --- |
|  | **Hub Registrants** | | **SIRE** | | **Hub Registrants** | | **SIRE** | | **Hub Registrants** | | **SIRE** | | **Hub Registrants** | | **SIRE** | |
|  | N | % | N | % | N | % | N | % | N | % | N | % | N | % | N | % |
| Mild | 1209 | 64 | 54 | 64 | 1172 | 62 | 52 | 62 | 892 | 47 | 29 | 35 | 1169 | 62 | 44 | 52 |
| Moderate | 532 | 28 | 18 | 21 | 313 | 17 | 14 | 17 | 214 | 11 | 14 | 17 | 466 | 25 | 18 | 21 |
| Severe | 139 | 7 | 12 | 14 | 406 | 22 | 18 | 21 | 786 | 42 | 41 | 49 | 255 | 14 | 22 | 26 |

**Figure 1S: Change in Mean Scores on Hub metrics for the Participants in the Mild, Moderate and Severe Groups of Response**

**
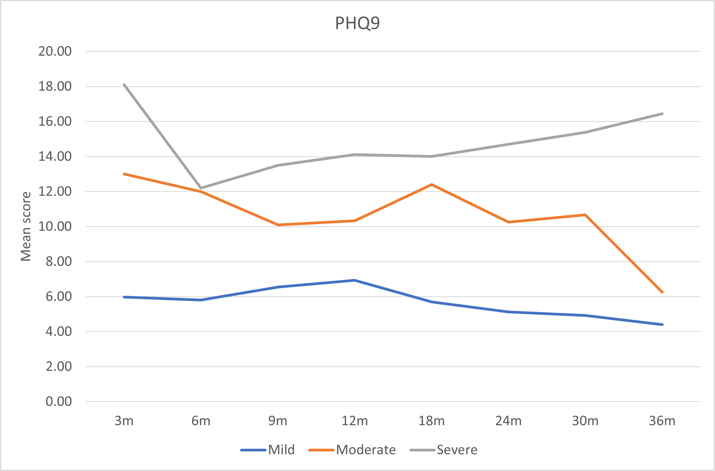

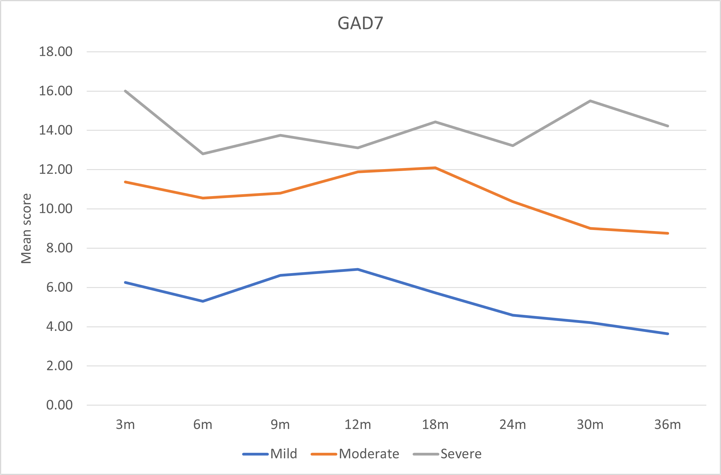
**

**
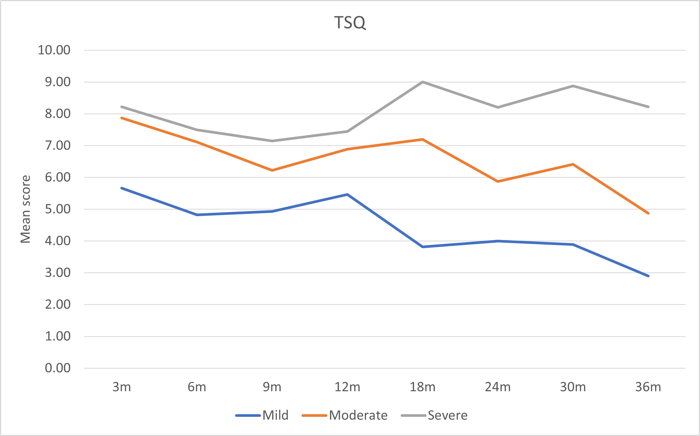

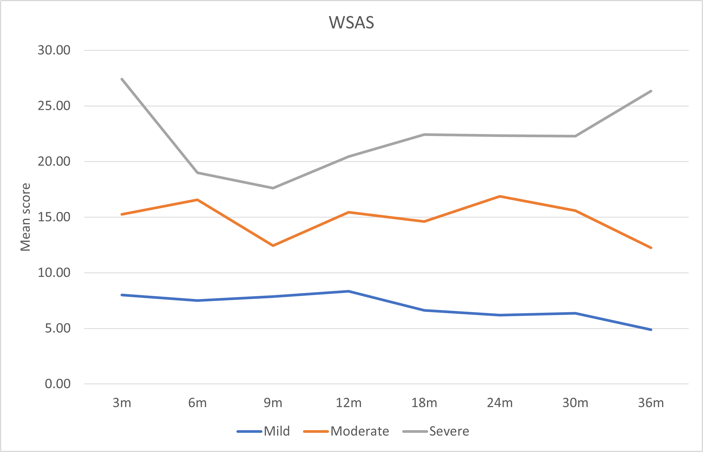
**

**Table 5S: Linear Regression for Mild, Moderate and Severe Groups in Hub scores over 3 years**

| **Measure** | **Pearson** | **R square** | **B** | **Lower 95% CI** | **Upper 95% CI** | **Sig (p)** |
| --- | --- | --- | --- | --- | --- | --- |
| **PHQ9** |  |  |  |  |  |  |
| Mild | -0.12 | 0.014 | -0.059 | -0.12 | 0.002 | 0.058 |
| Moderate | -0.27 | 0.073 | -0.127 | -0.228 | -0.026 | 0.014 |
| Severe | 0.018 | 0.000 | 0.011 | -0.141 | 0.164 | 0.882 |
| **GAD7** |  |  |  |  |  |  |
| Mild | -0.191 | 0.036 | -0.083 | -0.137 | -0.03 | 0.002 |
| Moderate | -0.18 | 0.033 | -0.078 | -0.175 | 0.018 | 0.109 |
| Severe | -0.002 | 0.000 | -0.001 | -0.122 | 0.12 | 0.987 |
| **TSQ** |  |  |  |  |  |  |
| Mild | -0.288 | 0.083 | -0.072 | -0.102 | -0.042 | <0.001 |
| Moderate | -0.266 | 0.071 | -0.057 | -0.103 | -0.01 | 0.018 |
| Severe | 0.122 | 0.015 | 0.024 | -0.025 | 0.073 | 0.337 |
| **WSAS** |  |  |  |  |  |  |
| Mild | -0.112 | 0.013 | -0.075 | -0.158 | 0.009 | 0.81 |
| Moderate | -0.051 | 0.003 | -0.029 | -0.161 | 0.102 | 0.657 |
| Severe | 0.074 | 0.005 | 0.062 | -0.153 | 0.278 | 0.565 |

**Table 6S: WEMWBS and Post-event Distress Scores and their Correlations with Hub Scores**

| **All Participants** (N=84) | | | | | |
| --- | --- | --- | --- | --- | --- |
| **Hub measures** | | **WEMWBS** | | **Post-event Distress** | |
|  |  | **Pearson Correlation** | **Significance (p)** | **Pearson Correlation** | **Significance (p)** |
| 3-month scores | PHQ9 | -0.394 | 0.001* | 0.646 | <0.001* |
|  | GAD7 | -0.283 | 0.017* | 0.677 | <0.001* |
|  | TSQ | -0.225 | 0.67 | 0.68 | <0.001* |
|  | WSAS | -0.433 | <0.001* | 0.684 | <0.001* |
| 6-month scores | PHQ9 | -0.326 | 0.01* | 0.512 | <0.001* |
|  | GAD7 | -0.237 | 0.064 | 0.562 | <0.001* |
|  | TSQ | -0.197 | 0.135 | 0.642 | <0.001* |
|  | WSAS | -0.285 | 0.03* | 0.599 | <0.001* |
| 36-month scores | PHQ9 | -0.638 | <0.001* | 0.42 | 0.003* |
|  | GAD7 | -0.647 | <0.001* | 0.476 | 0.001* |
|  | TSQ | -0.483 | 0.001* | 0.614 | <0.001* |
|  | WSAS | -0.596 | <0.001* | 0.526 | <0.001* |

**Table 7S: Mean Difference of Each Secondary Stressor Item and the Full-scale Scores**

| **Secondary Stressor Questions** | **N** | **Mean** | **SD** | **T Test** | **P** | **Mean difference from scale mean** | **95% CI** | | **SS Factor** |
| --- | --- | --- | --- | --- | --- | --- | --- | --- | --- |
|  |  |  |  |  |  |  | **Lower** | **Upper** |  |
| Loss of income | 84 | 1.94 | 1.33 | -4.75 | <0.001* | -0.69 | -0.98 | -0.40 | 3 |
| Loss or lack of employment | 84 | 1.88 | 1.28 | -5.35 | <0.001* | -0.75 | -1.03 | -0.47 | 3 |
| New or additional pressures on me at work | 83 | 2.63 | 1.45 | -0.02 | 0.982 | 0.00 | -0.32 | 0.31 | 3 |
| Difficulties with making an application to the compensation scheme | 84 | 2.10 | 1.63 | -3.00 | 0.004* | -0.53 | -0.89 | -0.18 | 4 |
| Difficulties with the unfairness of the compensation scheme | 84 | 2.12 | 1.64 | -2.83 | 0.005* | -0.51 | -0.87 | -0.16 | 4 |
| New or continuing physical health problems | 84 | 2.48 | 1.51 | -0.93 | 0.353 | -0.15 | -0.48 | 0.17 | 5 |
| New or continuing mental health problems | 84 | 3.67 | 1.13 | 8.38 | <0.001* | 1.04 | 0.79 | 1.28 | 1 |
| Lack of access to the physical healthcare I need | 84 | 1.75 | 1.03 | -7.84 | <0.001* | -0.88 | 0.30 | -0.66 | 5 |
| Lack of access to the mental healthcare I need | 83 | 2.47 | 1.40 | -1.04 | 0.300 | -0.16 | -0.47 | 0.15 | 2 |
| Difficulties with finding information and support | 83 | 2.46 | 1.17 | -1.34 | 0.184 | -0.17 | -0.43 | 0.08 | 2 |
| Lack of education opportunities or facilities | 84 | 1.86 | 1.16 | -6.09 | <0.001* | -0.77 | -1.03 | -0.52 | _ |
| Exposure to negative reports in the news media | 84 | 3.88 | 1.08 | 10.62 | <0.001* | 1.25 | 1.02 | 1.49 | _ |
| Social media | 82 | 3.55 | 1.20 | 6.94 | <0.001* | 0.92 | 0.66 | 1.18 | _ |
| The response from the community | 84 | 2.81 | 1.38 | 1.19 | 0.238 | 0.18 | -0.12 | 0.48 | 2 |
| Disruption to relationships with my friends | 83 | 2.88 | 1.30 | 1.75 | 0.084 | 0.25 | -0.03 | 0.53 | 1 |
| Disruption to relationships within my family | 83 | 2.81 | 1.27 | 1.27 | 0.208 | 0.18 | -0.10 | 0.46 | 1 |
| Disruption to my leisure and relaxation activities | 83 | 3.29 | 1.29 | 4.64 | <0.001* | 0.66 | 0.38 | 0.94 | 1 |
| Spending more time helping my family, friends or other people in the community | 84 | 2.79 | 1.28 | 1.11 | 0.268 | 0.16 | -0.12 | 0.43 | 1 |

**Table 8S: Pattern Factor Loadings for Exploratory Factor Analysis of Secondary Stressor Questions**

We conducted a principal axis factor analysis on the 18 items (varimax) in order to explore the extent to which the items in the secondary stressors scale clustered. The Kaiser-Meyer-Olkin (KMO) measure verified the sampling adequacy for the analysis, KMO=0.845.[42] Initial analysis provided eigenvalues for each factor in the data. Five factors had eigenvalues over Kaiser’s criterion of 1 and in combination explained 65% of the variance. We retained all five factors, though the scree plot did not clearly suggest five and only indicated two. Table 8S shows the factor loadings after rotation. The items that cluster on the same factor suggest that: Factor 1 indexes Families & Friends stressors (e.g., ‘Disruption to relationships with my friends/within my family’); Factor 2 reflects Work stressors (e.g., ‘Loss or lack of employment’); Factor 3 indexes issues with Compensation (e.g., Difficulties with making an application to the compensation scheme’); Factor 4 represents problems with Services (e.g., ‘Difficulties with finding information and support’/‘Lack of access to the mental healthcare I need’); and Factor 5 appears to represents stressors associated with Physical Health and accessing physical healthcare (e.g., ‘New or continuing physical health problems’).

| **Questionnaire items** | | **Factor 1**  **Families and Friends** | **Factor 2**  **Work** | **Factor 3**  **Compensation** | **Factor 4**  **Services** | **Factor 5**  **Physical Health** |
| --- | --- | --- | --- | --- | --- | --- |
| Q71 | Disruption to relationships with my friends | 0.949 |  |  |  |  |
| Q72 | Disruption to relationships within my family | 0.806 |  |  |  |  |
| Q73 | Disruption to my leisure and relaxation activities | 0.666 |  |  |  |  |
| Q74 | Spending more time helping my family, friends or other people in the community | 0.543 |  |  |  |  |
| Q63 | New or continuing mental health problems | 0.413 |  |  |  |  |
| Q68 | Exposure to negative reports in the news media |  |  |  |  |  |
| Q58 | Loss or lack of employment |  | 0.975 |  |  |  |
| Q57 | Loss of income |  | 0.824 |  |  |  |
| Q59 | New or additional pressures on me at work |  | 0.400 |  |  |  |
| Q69 | Social media |  |  |  |  |  |
| Q60 | Difficulties making an application to the compensation scheme |  |  | 0.817 |  |  |
| Q61 | Difficulties with the unfairness of the compensation scheme |  |  | 0.796 |  |  |
| Q66 | Difficulties with finding information and support |  |  |  | 0.866 |  |
| Q65 | Lack of access to the mental healthcare I need |  |  |  | 0.748 |  |
| Q70 | The response from the community |  |  |  | 0.669 |  |
| Q67 | Lack of education opportunities or facilities |  |  |  |  |  |
| Q62 | New or continuing physical health problems |  |  |  |  | 0.898 |
| Q64 | Lack of access to the physical healthcare I need |  |  |  |  | 0.482 |
| **Eigen values** | | **7.99** | **1.68** | **1.30** | **1.16** | **1.04** |
| **% of Variance** | | **42.6** | **7.89** | **6.08** | **4.65** | **4.13** |

Extraction Method: Principal Axis Factoring

Rotation Method: Oblimin with Kaiser Normalisation

*Note.* Loadings < .40 are omitted

**Table 9S: Associations of Secondary Stressors with Hub Response Categories**

| **Hub Categories** | ***Composite Score of All Secondary Stressors v Initial Hub Categories*** | | | | | | |
| --- | --- | --- | --- | --- | --- | --- | --- |
|  | **Mild** | | **Moderate** | | **Severe** | | **One-way Anova significance** |
| Baseline Hub Measures by Category at 3/6m | N=41 | 2.24 | N=21 | 2.76 | N=22 | 3.24 | P<0.001 |
| Final Hub Measures by Category at 30/36m | N=36 | 2.35 | N=12 | 2.91 | N=10 | 3.19 | P< 0.001 |

**Table 10S: Mean Social Cure Variables Scores for Participants Categorised as Showing Mild, Moderate and Severe Responses at Initial Hub Assessment**

| **Baseline Hub Scores by Category at 3/6 Months** | **Social Identity** | **Efficacy** | **Friends & Families NOT at the Arena** | **Arena Support** | **Support from One’s Workplace** |
| --- | --- | --- | --- | --- | --- |
| Mild (N=41) | 4.18 | 3.43 | 3.29 | 3.48 | 2.95 |
| Moderate (N=21) | 4.00 | 3.10 | 2.71 | 3.60 | 2.92 |
| Severe (N=22) | 3.96 | 2.71 | 2.62 | 3.41 | 2.76 |
| One-way ANOVA significance  Multiple comparisons if ANOVA significant (Tukey HSD) | 0.531 | 0.002*  Mild-Moderate 0.231  Mild-Severe 0.001*  Moderate-Severe 0.208 | 0.017*  Mild-Moderate 0.079  Mild-Severe 0.031*  Moderate-Severe 0.948 | 0.635 | 0.842 |

**Table 11S: Mean Social Cure Variables Scores for Participants Categorised as Mild,**

**Moderate and Severe Responses at Final Hub Assessment**

| **Final Hub Scores by Category at 30/36 Months** | **Social Identity** | **Efficacy** | **Families & Friends NOT at the Arena** | **Arena Support** | **Support from One’s Workplace** |
| --- | --- | --- | --- | --- | --- |
| Mild (N=41) | 4.16 | 3.28 | 3.17 | 3.48 | 2.90 |
| Moderate (N=21) | 4.11 | 3.17 | 2.56 | 3.56 | 2.72 |
| Severe (N=22) | 3.60 | 2.15 | 2.23 | 3.30 | 2.48 |
| One-way ANOVA significance  Multiple comparisons if ANOVA significant (Tukey HSD) | 0.207 | <0.001*  Mild-Moderate 0.881  Mild-Severe <0.001*  Moderate-Severe 0.003* | 0.007*  Mild-Moderate 0.099  Mild-Severe 0.011*  Moderate-Severe 0.667 | 0.687 | 0.643 |

**Table 12S: Correlations of Post-event Distress and Current Mental Wellbeing (WEMWBS) Scores with Secondary Stressors**

| **Secondary Stressors (N=84)** | **Families and Friends** | | **Services** | | **Work** | | **Compensation** | | **Physical Health** | |
| --- | --- | --- | --- | --- | --- | --- | --- | --- | --- | --- |
| Mean (SD) | 3.090 (1.020) | | 2.579 (1.144) | | 2.149 (1.149) | | 2.107 (1.595) | | 2.113 (1.168) | |
|  | Pearson | P | Pearson | P | Pearson | P | Pearson | P | Pearson | P |
| Post-event Distress | 0.681 | <0.001* | 0.530 | <0.001* | 0.548 | <0.001* | 0.444 | <0.001* | 0.468 | <0.001* |
| WEMWBS | -0.363 | 0.001* | 0.096 | 0.403 | 0.017 | 0.886 | -0.075 | 0.516 | -0.161 | 0.160 |
